# Structural analysis of dual specificity phosphatases, the only type of protein tyrosine phosphatases found in humans and across diverse microorganisms

**DOI:** 10.71150/jm.2506006

**Published:** 2025-10-31

**Authors:** Bonsu Ku

**Affiliations:** Orphan Disease Therapeutic Target Research Center, Korea Research Institute of Bioscience and Biotechnology, Daejeon 34141, Republic of Korea

**Keywords:** dual specificity phosphatase, DUSP, protein tyrosine phosphatase, PTP, structure, TpbA, Tk-PTP, SsoPTP, VH1

## Abstract

Dual specificity phosphatases (DUSPs), a subfamily of the protein tyrosine phosphatase (PTP) family, dephosphorylate not only phosphotyrosine but also phosphoserine and phosphothreonine residues. Beyond the 26 members of this family in humans, DUSPs represent the only type of PTPs found across a wide range of microorganisms, including bacteria, archaea, and viruses. This review presents a comprehensive structural analysis of human and microbial DUSPs. These proteins commonly share core features, such as a typical DUSP fold, shallow active site pocket, signature active site motif known as the P-loop, and conserved aspartate residue that acts as a general acid/base. However, DUSPs from diverse microorganisms also display unique structural and functional characteristics. *Pseudomonas aeruginosa* TpbA is the only bacterial DUSP identified to date, while a second candidate was proposed in this review. Archaeal DUSPs are hyperthermostable, contain a unique motif in their P-loops, and employ dual general acid/base residues. Poxviral DUSPs are characterized by the formation of domain-swapped homodimers. The presence of DUSPs across all domains of life and viruses, along with their low specificity for phosphorylated amino acids and structural similarity to classical PTPs, suggests that DUSPs represent the ancestral form of PTPs.

## Introduction

Phosphatase-mediated reversible dephosphorylation is a major post-translational modification of proteins. By removing phosphate groups from the side chains of specific amino acids, these enzymes control the activity, localization, and interactions of their substrate proteins, thereby regulating numerous biological processes, including cell cycle progression, signal transduction, apoptosis, and differentiation (Ardito et al., 2017; Tarrant and Cole, 2009; Ubersax and Ferrell, 2007). Dephosphorylation primarily occurs on the phosphorylated side chains of three amino acids: serine and threonine, which are targeted by serine/threonine phosphatases, and tyrosine, which is dephosphorylated by protein tyrosine phosphatases (PTPs). More than a hundred PTP proteins have been identified in the human genome, emphasizing the importance of controlling tyrosine dephosphorylation (Lee et al., 2015; Tonks, 2006). The human PTP family comprises several subgroups, including receptor-type and nonreceptor-type classical PTPs, which are specific for phosphotyrosine, and dual specificity phosphatases (DUSPs), which are characterized by their ability to dephosphorylate both phosphoserine/threonine and phosphothyrosine residues (Jeong et al., 2014; Patterson et al., 2009).

PTPs are found in a wide range of microorganisms, including bacteria (Kuban-Jankowska et al., 2022; Standish and Morona, 2014; Whitmore and Lamont, 2012), archaea (Esser et al., 2016; Kennelly, 2014; Stravopodis and Kyrpides, 1999), and viruses (Guan and Dixon, 1993). They contribute to microbial survival and environmental adaptation through various mechanisms. Several bacterial PTPs, such as VpsU from *Vibrio cholerae*, Wzb from *Escherichia coli*, and CpsB from *Streptococcus pneumoniae*, play pivotal roles in regulating the biosynthesis of capsular and extracellular polysaccharides that function as key virulence factors (Standish and Morona, 2014). SptP from *Salmonella enterica* dephosphorylates the host AAA+ ATPase protein p97 to promote intracellular bacterial replication within *Salmonella*-containing vacuoles (Humphreys et al., 2009). PtpA from *Mycobacterium tuberculosis* dephosphorylates human vacuolar protein sorting 33B to inhibit phagosome-lysosome fusion (Bach et al., 2008). Similarly, H1 from *Vaccinia* virus dephosphorylates signal transducer and activator of transcription 1 (STAT1) to interfere with the host antiviral immune response (Najarro et al., 2001). Notably, all PTPs identified and characterized using structural and biochemical approaches in archaea (Chu and Wang, 2007; Pinkston et al., 2021; Yun et al., 2018) and viruses (Cui et al., 2023; Koksal et al., 2009; Phan et al., 2007; Segovia et al., 2017) are classified as DUSPs. DUSPs are also present in bacteria (Koveal et al., 2013; Xu et al., 2015), making them the only type of PTPs found in diverse microorganisms and highlighting their potential functional significance.

This review presents an in-depth structural analysis of DUSP proteins and describes their similarities and differences at the molecular level, including those of 26 human DUSPs (Tables 1 and 2) and seven microbial DUSPs (Table 3) comprising one bacterial DUSP (TpbA), two archaeal DUSPs (Tk-PTP and SsoPTP), and four poxviral DUSPs. While several human DUSPs contain an accessory domain in addition to the catalytic domain, such as the rhodanese domain found in DUSP6, DUSP10, and DUSP16 (Farooq et al., 2001; Tao and Tong, 2007; Zhang et al., 2011), all identified microbial DUSPs possess a single catalytic domain. Therefore, this review focuses on the structural analysis of the phosphatase domain of DUSP proteins.

## Structural Analysis of the Catalytic Domain of Human DUSPs

### Overall structural features of human DUSPs

Human DUSPs can be categorized into two groups: mitogen-activated protein kinase phosphatase (MKP)-type and atypical DUSPs. MKP-type DUSPs, including DUSP1, DUSP2, DUSP4, DUSP5, DUSP6, DUSP7, DUSP8, DUSP9, DUSP10, DUSP14, and DUSP16 (Table 1), dephosphorylate the mitogen-activated protein kinases (MAPKs) and suppress their activities, including extracellular signal-regulated kinases (ERKs), c-Jun N-terminal kinases (JNKs), and p38 kinases (Braicu et al., 2019; Patterson et al., 2009). Based on their subcellular localization and substrate specificity, MKP-type DUSPs are further divided into three subgroups: nuclear MKPs (DUSP1, DUSP2, DUSP4, and DUSP5), ERK-selective cytoplasmic MKPs (DUSP6, DUSP7, and DUSP9), and JNK/p38-selective MKPs (DUSP8, DUSP10, and DUSP16) (Caunt and Keyse, 2013; Patterson et al., 2009). The remaining members are classified as atypical DUSPs (Table 2): DUSP3, DUSP11, DUSP12, DUSP13a, DUSP13b, DUSP15, DUSP18, DUSP19, DUSP21, DUSP22, DUSP23a, DUSP23b, DUSP26, DUSP28, and DUSP29 (Patterson et al., 2009). Structural studies have revealed the three-dimensional structure of the phosphatase domain in most human DUSPs, except for DUSP21 and DUSP23b, providing insights into the biochemical basis of their functions (Tables 1 and 2).

**Fig. 1.**
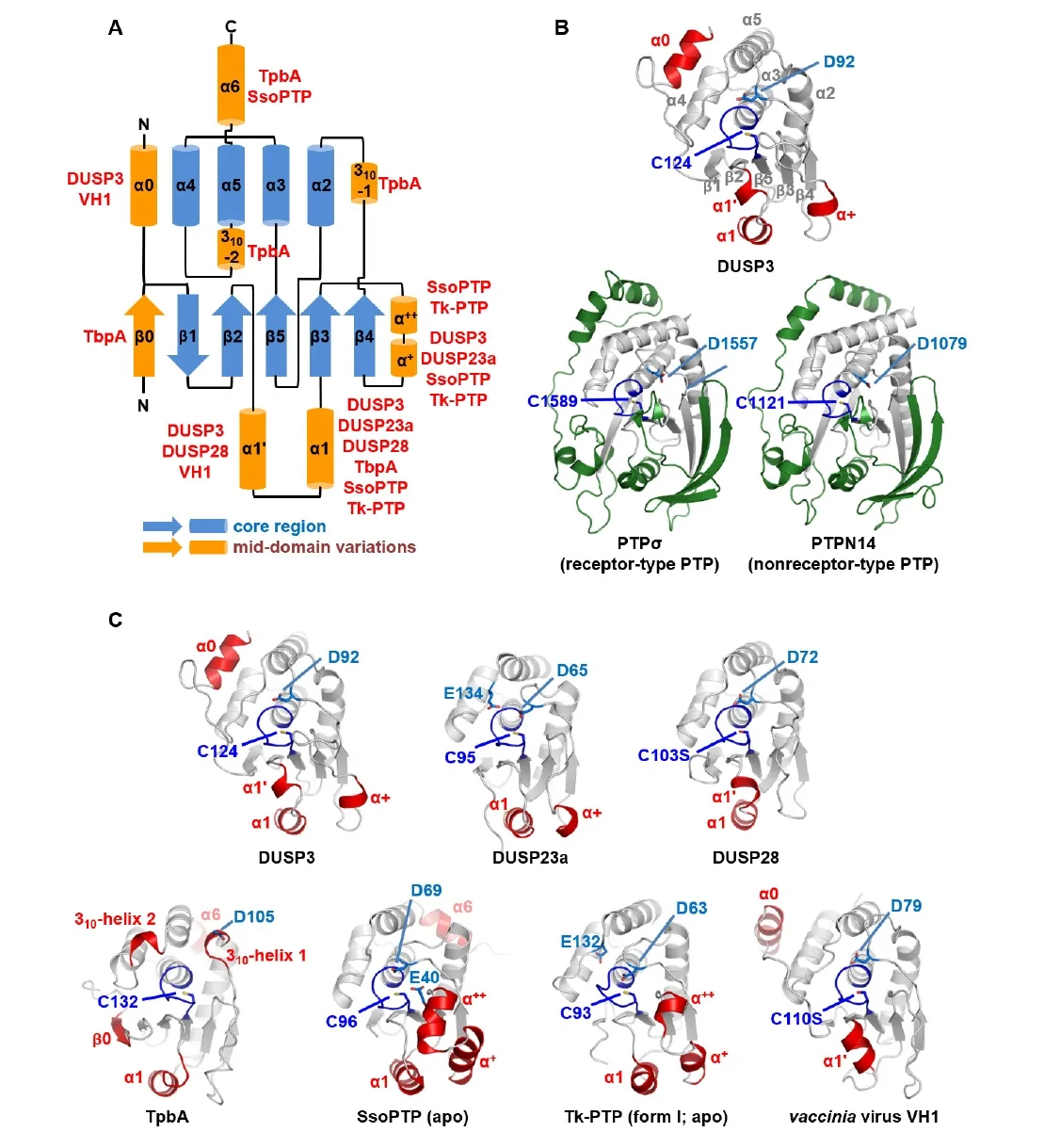
Overall fold of DUSP proteins. (A) Schematic representation of the secondary structural elements of DUSPs. The core region components are shown in blue, whereas mid-domain variations are highlighted in orange. Red labels adjacent to each mid-domain variation indicate the DUSP proteins in which these components are found. For clarity, only the seven DUSPs depicted in Fig. 1C are labeled. The numbering of the secondary structural elements follows the previous structural study of human DUSP (Jeong et al., 2014). (B) Overall fold of the catalytic domains of three representative PTP proteins. The region corresponding to the DUSP core is shown in white, whereas the remaining portions are highlighted in red for DUSP3 or in green for PTPσ and PTPN14. The secondary structural elements of DUSP3 are labeled. In all three structures, the P-loop motifs are colored in blue, and the catalytic residues (blue) and general base/acid residues (cyan) are shown as sticks and labeled. Structural models were generated using PyMOL. The PDB codes are as follows: 1VHR for DUSP3, 2FH7 for PTPσ, and 6IWD for PTPN14. (C) Overall fold of three human and four microbial DUSP proteins. The region corresponding to the DUSP core is shown in white, whereas the remaining portions are highlighted and labeled in red. In all seven structures, the P-loop motifs are colored in blue, and the catalytic residues (blue) and general base/acid residues (cyan) are shown as sticks and labeled. The catalytic cysteine residues of DUSP28 and *vaccinia* virus VH1 were substituted with serine, which eliminates catalytic activity that can hinder protein crystallization and prevents accidental oxidation of cysteine thiolate during protein purification and crystallization. Structural models were generated using PyMOL. The PDB codes are as follows: 1VHR for DUSP3, 4ERC for DUSP23a, 5Y15 for DUSP28, 4R0S for TpbA, 2I6I for SsoPTP (apo), 5Z59 for Tk-PTP (form I; apo), and 3CM3 for *vaccinia* virus VH1.

Their phosphatase domain adopts a typical “DUSP” fold, comprising a core region that contains a five-stranded central β-sheet surrounded by four α-helices, as well as several mid-domain variations (Jeong et al., 2014). Although the core region is conserved across all DUSP proteins and forms a structurally homologous backbone, mid-domain variations differ among DUSP members. These features contribute to structural diversity that may influence substrate specificity and interactions with other proteins (Fig. 1A). Interestingly, the region corresponding to the DUSP core is conserved not only among DUSPs, but also in the catalytic domain of classical PTPs, such as PTPσ, a receptor-type PTP, and PTPN14, a nonreceptor-type PTP (Almo et al., 2007; Yun et al., 2019), suggesting that DUSPs and classical PTPs have shared a common structural backbone throughout evolution (Fig. 1B and 1C).

**Fig. 2.**
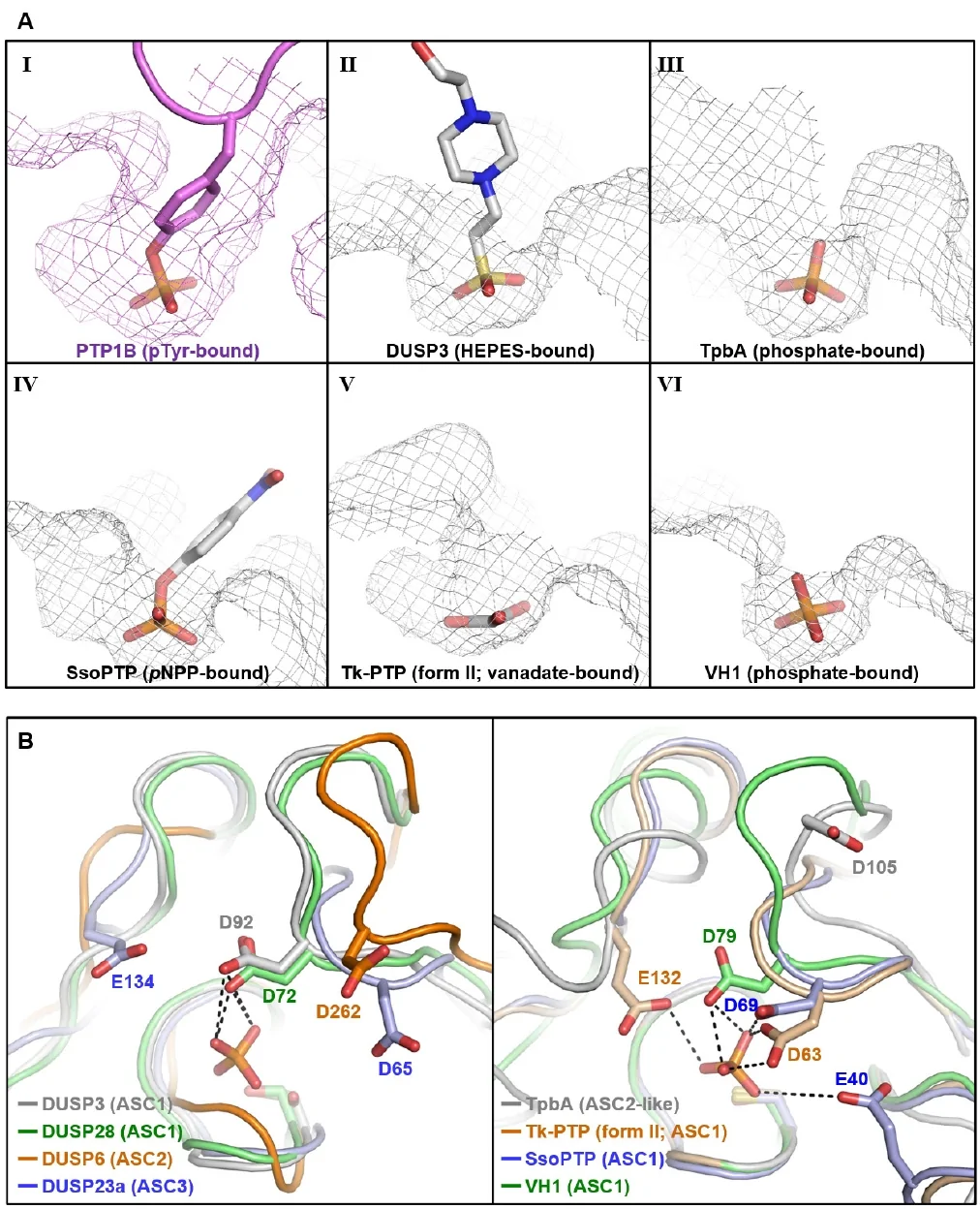
Active site architecture of DUSP proteins. (A) Active site pocket regions of PTP proteins shown as surface mesh representations. PTP1B, a nonreceptor-type classical PTP, is highlighted in violet, whereas DUSP proteins are shown in gray. To clearly illustrate the depth of the catalytic pockets, phosphotyrosine (for PTP1B) or its mimetics (HEPES for DUSP3, phosphate ion for TpbA and *vaccinia* virus VH1, *p*NPP for SsoPTP; and vanadate for Tk-PTP) are displayed as sticks along with the protein structures. Structural models were generated using PyMOL. The PDB codes are as follows: 4ZRT for PTP1B, 1VHR for DUSP3, 4R0S for TpbA, 2I6I for *p*NPP-bound SsoPTP, 5Z5A for vanadate-bound Tk-PTP (form II), and 3CM3 for *vaccinia* virus VH1. (B) ASC types. Four human (left) and four microbial (right) DUSP proteins are structurally aligned. Catalytic and general acid/base residues are shown as sticks; for clarity, only the general acid/base residues are labeled. Phosphate ions from the DUSP28 (left) or TpbA (right) structures are included to illustrate their association with the general acid/base aspartate residue (indicated by dashed lines), which determines the ASC types. Structural models were generated using PyMOL. The PDB codes are as follows: 1VHR for DUSP3, 5Y15 for DUSP28, 1MKP for DUSP6, 2IMG for DUSP23a, 4R0S for TpbA, 5Z5A for Tk-PTP (form II), 2I6I for SsoPTP (apo), and 3CM3 for *vaccinia* virus VH1.

Another common structural feature of human DUSPs is their shallow active site pocket. In the common catalytic mechanism of PTPs, a phosphate moiety is cleaved from a phosphorylated tyrosine residue through the coordinated action of three key residues: a catalytic cysteine that acts as a nucleophile, an arginine that anchors the phosphate group, and an aspartate that functions as a general acid/base residue (Selner et al., 2014; Tautz et al., 2013). Although this triad can also remove phosphate groups from phosphoserine and phosphothreonine, most classical PTPs exhibit strict specificity for phosphotyrosine. This specificity is largely attributed to their deep and narrow catalytic pockets, which sterically hinder access to phosphorylated serine or threonine residues (Fig. 2A, panel I). In contrast, human DUSPs, such as DUSP3, possess a shallow and broad catalytic pocket (Fig. 2A, panel II), which enables dephosphorylation of all three types of phosphorylated residues to function as “dual specificity” phosphatases.

### Catalytic activity determinants: P-loop and general acid/base residue

**Fig. 3.**
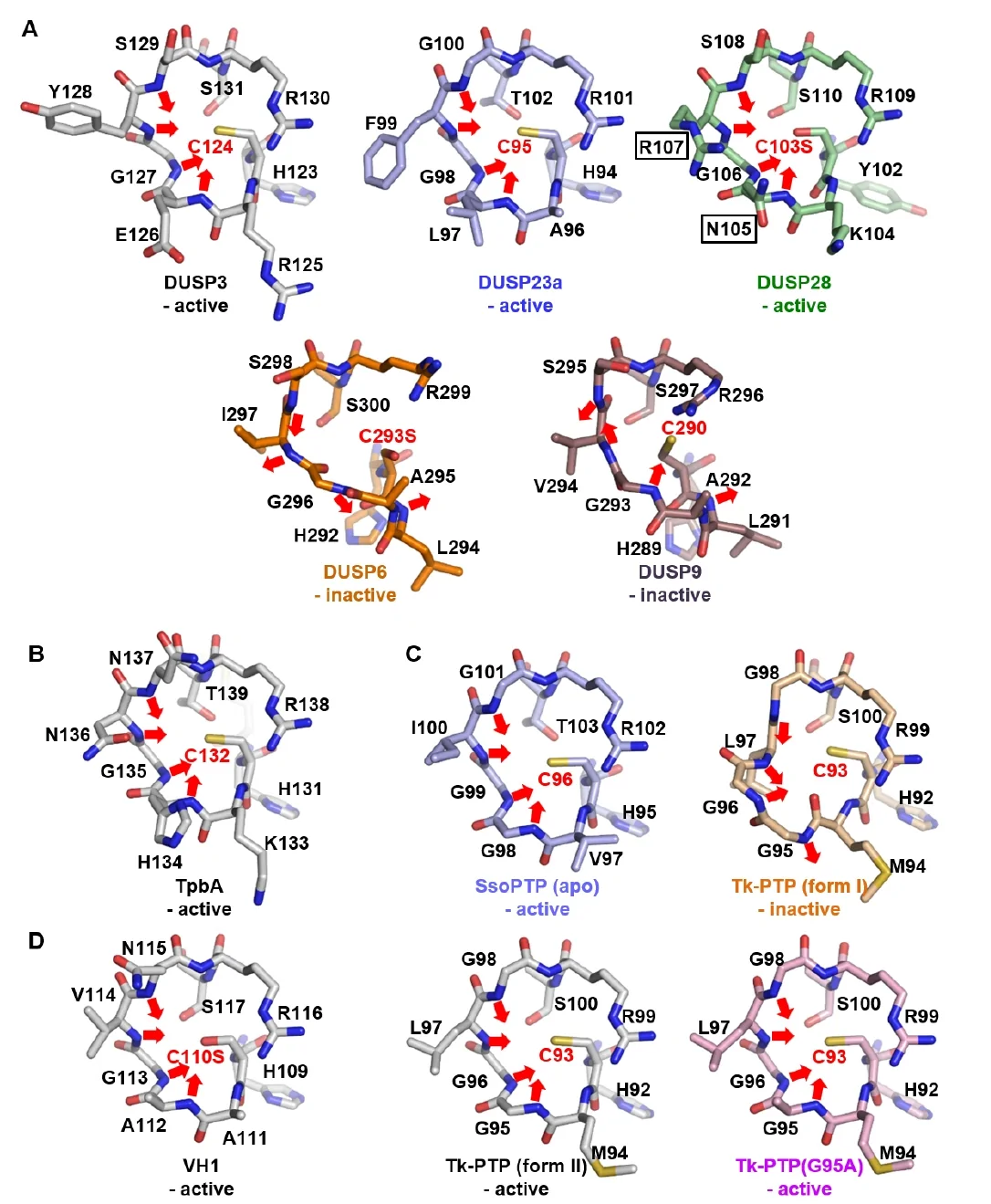
P-loop conformations of DUSP proteins. The P-loops of five human DUSPs (A), TpbA (B), two archaeal DUSPs (C), and VH1 (D) are shown as sticks for comparison. Catalytic residues are labeled in red, whereas other residues are labeled in black. Arrows indicate the orientation of the main chain amides of the four central residues of the P-loop. Asn105 and Arg107 in DUSP28, which regulate substrate accessibility, are also emphasized by boxes. The catalytic cysteine residues of DUSP6, DUSP28, and *vaccinia* virus VH1 were substituted with serine, as described in the legend of Fig. 1C. Structural models were generated using PyMOL. The PDB codes are as follows: 1VHR for DUSP3, 4ERC for DUSP23a, 5Y15 for DUSP28, 1MKP for DUSP6, 3LJ8 for DUSP9, 4R0S for TpbA, 2I6I for SsoPTP (apo), 5Z59 for Tk-PTP (form I), 5Z5A for Tk-PTP (form II), 5Z5B for Tk-PTP(G95A), and 3CM3 for *vaccinia* virus VH1.

Most PTP proteins, including DUSPs, contain a signature active site motif (H-C-x-x-G-x-x-R-S/T), named the phosphate-binding loop, or the P-loop (Tabernero et al., 2008; Tautz et al., 2013). This motif possesses a catalytic cysteine residue serving as a nucleophile and a conserved arginine residue anchoring the phosphate group of the substrate during the dephosphorylation reaction. Structural and biochemical investigations revealed a correlation between the main or side chain arrangement of the P-loop and phosphatase activity of DUSP proteins. In most human DUSP protein structures, the P-loop (H-C-x-x-G-x-x-R-S/T) adopts a catalytically active conformation, where the main chain backbone amides are oriented toward the active site pocket and create a positive electrostatic potential together with the guanidinium group of the conserved arginine residue and dipole moment from α3 following the P-loop (Fig. 3A, top). This positive electrostatic potential plays a crucial role in the enzymatic reaction, not only by lowering the p*K*a value of the catalytic cysteine residue to stabilize it in the thiolate state but also by neutralizing the negative charge of the substrate phosphate group (Kolmodin and Aqvist, 2001; Zhang, 2002; Zhang et al., 1994). In contrast, at least two human DUSPs, DUSP6 (MKP-3) and DUSP9 (MKP-4), contain a distorted P-loop (Fig. 3A, bottom). In biochemical analyses, DUSP6 and DUSP9 exhibited very weak dephosphorylation activities, which were upregulated by interactions with MAPK proteins (Fjeld et al., 2000; Hong et al., 2005; Zhou and Zhang, 1999). Therefore, the binding of MAPK proteins to DUSP6, DUSP9, or other MKP subgroup members is thought to induce conformational changes in their P-loop region into a catalytically reactive form, resulting in activation of these DUSPs (Kondoh and Nishida, 2007).

The side chains of the P-loop can also regulate enzymatic activity by restricting substrate access to the active site pocket. Although the main chain portion of the P-loop in DUSP28 is well-arranged (Fig. 3A, top right), its enzymatic activity was exceptionally low (Ku et al., 2017). This is because the active site pocket is only partially exposed due to the presence of bulkier side chains at the fourth (Asn105) and sixth (Arg107) positions of the P-loop (Y-C-K-N^105^-G-R^107^-S-R-S) (Fig. 3A, top right). These positions are commonly occupied by smaller residues, such as alanine/valine (in 20 of 26 human DUSPs; at the fourth position) or valine/leucine/isoleucine (in 21 of 26 human DUSPs; at the sixth position). When N105A and R107V substitutions were introduced into DUSP28, its enzymatic activity for phosphotyrosine, phosphothreonine, and the artificial PTP substrate 6,8-difluoro-4-methylumbelliferyl phosphate (DiFMUP) was significantly enhanced (Ku et al., 2017), demonstrating that the P-loop side chain portions play a regulatory role in the catalytic activity of DUSP proteins.

In addition to the catalytic cysteine residue in the P-loop motif, a highly conserved aspartate residue, known as the general acid/base residue, is a key determinant of the dephosphorylation activity of PTP proteins. During the initial step of the DUSP-mediated enzymatic reaction, the side chain carboxylic acid of the conserved aspartate residue serves as a general acid by donating a proton to the Oη atom of the targeted phosphotyrosine residue. In subsequent steps, the deprotonated side chain carboxylate acts as a general base, activating a water molecule to hydrolyze the phosphoenzyme intermediate (Tautz et al., 2013). All human DUSPs, except for DUSP23a (Kuznetsov and Hengge, 2013), utilize an aspartate residue as the general acid/base. DUSP23a uses Glu134 as the primary general acid/base residue rather than Asp65 at a typical position (Fig. 2B, left). The general acid/base aspartate residue is in the β4−α2 loop (also known as the D-loop), whose arrangement determines the active site conformation (ASC) of DUSP proteins. Among the three ASC types, ASC1 adopts a closed state, whereas ASC2 and ASC3 represent open states (Jeong et al., 2014), because the general acid/base aspartate residue is oriented toward the phosphate moiety targeted for dephosphorylation only in ASC1 (Fig. 2B, left).

## Structural Analysis of Microbial DUSPs

### Structural analysis of bacterial DUSPs

Some bacteria encode PTPs in their genomes, which can be broadly classified into three groups: low molecular weight (LMW)-PTPs, polymerase and histidinol family of phosphoesterases, and eukaryotic-like phosphatases (Standish and Morona, 2014; Whitmore and Lamont, 2012). Tyrosine phosphatase related to biofilm formation A (TpbA) from *Pseudomonas aeruginosa*, a member of the eukaryotic-like phosphatase group, is the only bacterial PTP protein identified as a DUSP based on structural and biochemical analyses (Koveal et al., 2013; Xu et al., 2015). Although SP-PTP, a virulence factor of *Streptococcus pyogenes*, was reported to exhibit dephosphorylating activity toward both tyrosine-phosphorylated and serine/threonine-phosphorylated proteins (Kant et al., 2015; Kant and Pancholi, 2021), structural and biochemical analyses demonstrated that this protein is a phosphotyrosine-specific LMW-PTP rather than a DUSP (Ku et al., 2016). Therefore, SP-PTP will not be discussed further in this review.

*P. aeruginosa* is an aerobic, Gram-negative, non-spore forming, rod-shaped bacterium that is widely found in the environment, including soil and water (Diggle and Whiteley, 2020). This bacterium infects various hosts, such as plants, nematodes, insects, and mammals. In humans, it is an opportunistic pathogen that causes acute or chronic infections in the blood, respiratory tract, urinary tract, or other sites in immunocompromised individuals, and is a leading cause of mortality among patients with cystic fibrosis (Diggle and Whiteley, 2020). To successfully establish colonization and evade the host immune system, *P. aeruginosa* forms biofilms, which are crucial in the development of chronic infection caused by this bacterium (Wang et al., 2023). TpbA, the DUSP protein of *P. aeruginosa*, is a key regulatory factor in biofilm formation: once secreted into the periplasm, this bacterial phosphatase dephosphorylates and thereby inactivates its substrate protein TpbB, a diguanylate cyclase that catalyzes the synthesis of 3′,5′-cyclic diguanylic acid. As a result, the intracellular concentration of this second messenger is reduced, leading to suppression of *pel* operon transcription and consequent repression of biofilm formation (Pu and Wood, 2010). Because of its essential role, TpbA is considered a putative drug target against *P. aeruginosa*.

To date, three structures of TpbA have been reported: one (PDB code: 2M3V) was determined by nuclear magnetic resonance spectroscopy (Koveal et al., 2013), whereas the other two (PDB codes: 4R0S for the wild-type and 4R0T for the C132S mutant) were determined using X-ray crystallography (Xu et al., 2015) (Table 3). All three structures revealed that TpbA consists of a single catalytic phosphatase domain, containing the typical DUSP fold core region (α2–α5 and β1–β5) and several mid-domain variations, including β0, α1, two 3_10_-helices, and α6 (Fig. 1C). The active site pocket of this bacterial DUSP protein is shallow (Fig. 2A, panel III), consistent with a previous report showing that TpbA dephosphorylates the phosphotyrosine and phosphoserine/threonine residues of TpbB (Pu and Wood, 2010). The well-organized P-loop (H^131^-C-K-H-G-N-N-R-T^139^) of TpbA adopts a catalytically active conformation (Fig. 3B). Asp105, the general acid/base residue of TpbA, is oriented away from the catalytic cysteine in an ASC2-like manner, suggesting that TpbA was crystallized in an open conformation (Fig. 2B, right).

**Fig. 4.**
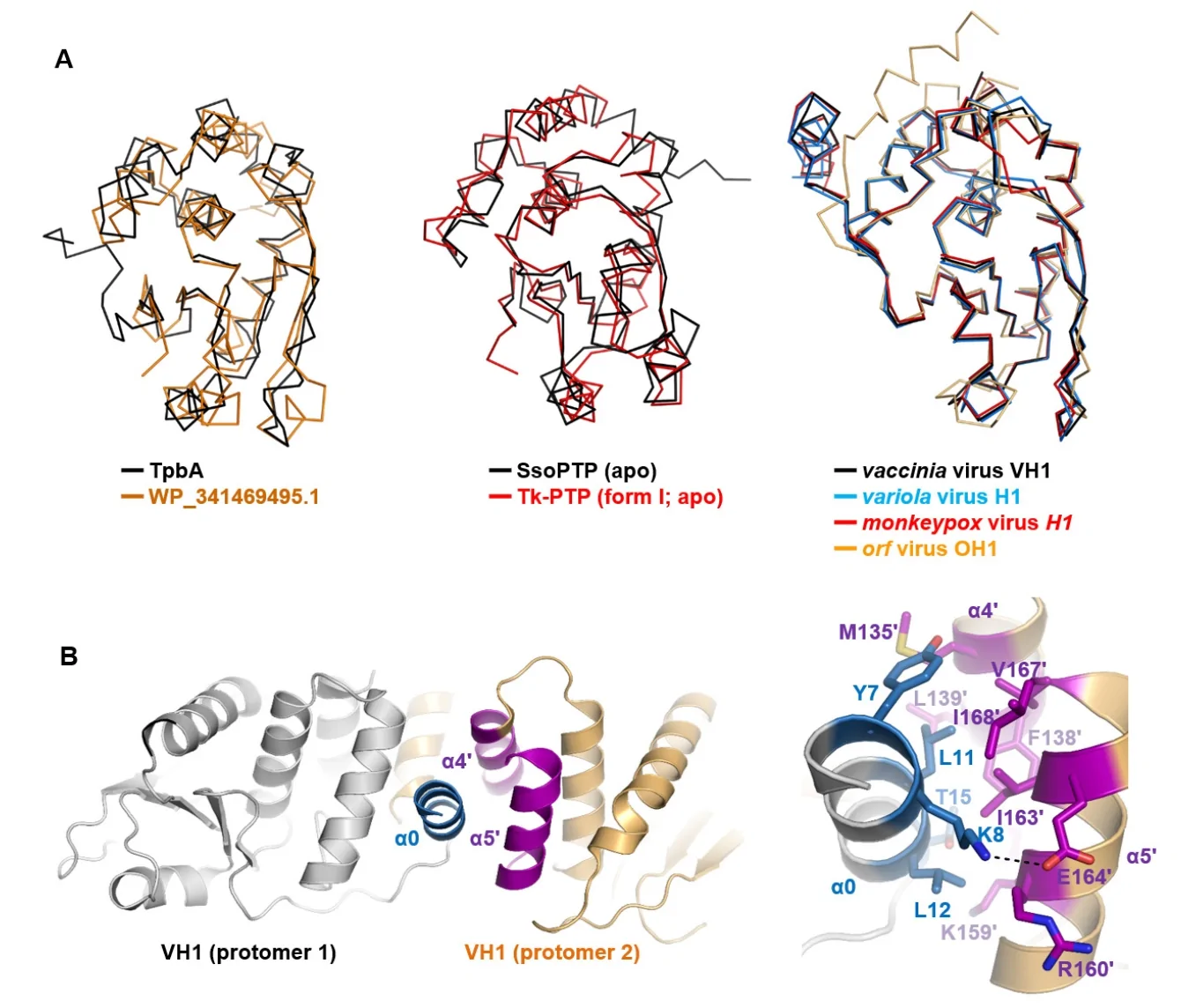
Structural analysis of microbial DUSP proteins. (A) Structural alignment of microbial DUSP proteins shown in ribbon presentation. (left) TpbA and a putative DUSP protein of *Ca. Chlorohelix. allophototropha* (RefSeq: WP_341469495.1). (middle) Apo forms of SsoPTP and Tk-PTP. (right) Four poxviral DUSP proteins. Structural models were generated using PyMOL. The PDB codes are as follows: 4R0S for TpbA, 2I6I for SsoPTP (apo), 5Z59 for Tk-PTP (form I), 3CM3 for *vaccinia* virus VH1, 2P4D for *variola* virus H1, 8GZ4 for *monkeypox* virus H1, and 5NCR for *orf* virus OH1. The three-dimensional structure of the *Ca. Chlorohelix allophototropha* DUSP candidate was modeled using AlphaFold 3. (B) Homodimerization of VH1. (left) Overall view of the dimer. The three α-helices involved in dimerization are labeled. (right) Atomic details of the dimer interface. Residues involved in dimerization are shown as sticks and labeled. For clarity, residues and secondary structure elements from the second protomer are marked with prime (') symbols. A dashed line indicates an electrostatic interaction. Structural models were generated using PyMOL. The PDB code is 3CM3 for *vaccinia* virus VH1.

To identify additional bacterial DUSPs, homology searches were conducted using the Basic Local Alignment Search Tool (BLAST; https://blast.ncbi.nlm.nih.gov/Blast.cgi). My attempts to find TpbA homologues led to the identification of 25 putative bacterial DUSPs. These candidates share 52.0–67.8% sequence identity with TpbA and retain at least six of the nine residues in the P-loop motif (H-C-x-H-G-x-x-R-T) (Table S1). However, all of these proteins were found in bacteria belonging to the phylum *Pseudomonadota* (Table S1). No TpbA homologues have been detected in the genomes of several representative model bacterial species, including *E. coli*, *Staphylococcus aureus*, and *Bacillus subtilis*. Nonetheless, a novel putative bacterial DUSP from outside the phylum *Pseudomonadota* was identified in the NCBI database (RefSeq: WP_341469495.1). This protein, which contains a conserved P-loop motif (H^96^-C-R-A-G-Y-G-R-T^104^), is encoded by the genome of *Candidatus Chlorohelix allophototropha*, belonging to the family *Candidatus Chloroheliaceae*, order *Candidatus Chloroheliales*, class *Chloroflexia*, and phylum *Chloroflexota*. Its three-dimensional structure, modeled by AlphaFold 3 (https://alphafoldserver.com), exhibited a canonical DUSP fold. This structure superimposed well on the TpbA structure with a root mean square deviation value of 1.97 Å over 128 aligned residues (Fig. 4A, left). Further structural and biochemical studies are required to determine whether this protein functions as a bacterial DUSP. Additional bacterial DUSPs may exist in lineages outside the phylum *Pseudomonadota*, which requires further investigation.

### Structural analysis of archaeal DUSPs

PTPs have also been identified in several species of archaea, a group of unicellular microorganisms that are genetically, biochemically, and ecologically distinct from bacteria (Esser et al., 2016; Kennelly, 2014; Stravopodis and Kyrpides, 1999). The three-dimensional structures of two archaeal PTP proteins were determined using X-ray crystallography: Tk-PTP from *Thermococcus kodakarensis* (Yun et al., 2018) and SsoPTP from *Sulfolobus solfataricus* (Chu and Wang, 2007; Pinkston et al., 2021), both belonging to the DUSP family. *T. kodakarensis* and *S. solfataricus* are hyperthermophilic archaea that were isolated in 1994 from a solfatara near Kodakara Island, Japan, and in 1980 from a hot spring in the Solfatara volcano, Italy, respectively (Morikawa et al., 1994), suggesting that these archaeal PTP proteins are thermostable.

The crystal structures of archaeal DUSP proteins were determined in several forms. The three-dimensional structures of SsoPTP were resolved by Chu and Wang (2007) in the apo (PDB code: 2I6I), phosphate-bound (PDB code: 2I6J), tungstate-bound (PDB code: 2I6M), *p*-nitrophenyl phosphate (*p*-NPP)-bound C96S mutant (PDB code: 2I6P), and two phosphopeptide-bound C96S mutant forms (PDB codes: 2I6O and 2DXP). Subsequently, two additional structures of SsoPTP bound to vanadate (PDB code: 7MPC) or 2-chloroethylsulfonate (PDB code: 7MPD) were reported (Pinkston et al., 2021) (Table 3). The crystal structures of Tk-PTP were determined in three forms: ligand-free wild-type (form I; PDB code: 5Z59), vanadate-bound and heat-treated wild-type (form II; PDB code: 5Z5A), and the G95A mutant (PDB code: 5Z5B) (Yun et al., 2018) (Table 3). Superimposition of SsoPTP and Tk-PTP revealed good alignment (Fig. 4A, middle), and both adopt the conventional DUSP fold, consisting of a core region (α2–α5 and β1–β5) and mid-domain variation components (α1 and two α-helices between β3 and β4 in both proteins, with an additional α6 helix in SsoPTP; Fig. 1C) along with a shallow active site pocket (Fig. 2A, panels IV and V). However, structural analyses showed that archaeal DUSPs have several unconventional features. Tk-PTP exhibited hyperthermostability, with a melting temperature of 86°C, which was substantially higher than those of human DUSP proteins such as DUSP3 (52°C) and DUSP28 (55°C) (Yun et al., 2018). Structural analysis revealed that the interior protein packing of Tk-PTP accounts for its hyperthermostability. Despite the high degree of overall structural similarity, the number of side chain-mediated intracellular carbon–carbon contacts (< 4.5 Å) per amino acid residue was 4.30 in Tk-PTP, significantly higher than those in DUSP3 (3.04) and DUSP28 (2.97) (Table 4). Although the thermostability of SsoPTP has not been experimentally measured, it is considered hyperthermostable because *S. solfataricus* is a well-known hyperthermophilic archaeon. This conclusion is further supported by the number of side chain-mediated intracellular carbon–carbon contacts (3.93 per residue), which is comparable to that in Tk-PTP (Table 4).

In the ligand-free wild-type Tk-PTP structure (form I), the P-loop (H^92^-C-M-G-G-L-G-R-S^100^) exhibited a noncatalytic distorted arrangement, in which the main chain backbone amides were not oriented toward the active site pocket (Fig. 3C, right top). This feature was also observed in DUSP6 and DUSP9 (Fig. 3A, bottom), indicating an inactive conformation. In contrast, the P-loop adopted an active conformation (form II) when this protein was heat-treated at 60°C for 3 h and subsequently incubated with sodium vanadate, a PTP inhibitor that targets the catalytic site pocket, prior to crystallization (Fig. 3C, left bottom). These structural analyses indicate that Tk-PTP oscillates between inactive and active conformations, presumably depending on the temperature conditions or substrate engagement. The presence of the “GG” motif, consisting of Gly95 and Gly96, is considered to confer such structural flexibility to the P-loop of Tk-PTP. Indeed, in the crystal structure of the G95A mutant form, the P-loop was well-ordered as an active conformation, even though the recombinant protein was not subjected to heat or sodium vanadate treatment before crystallization (Fig. 3C, right bottom). Consistently, phosphatase activity assays demonstrated that the active conformation-trapped G95A mutant Tk-PTP exhibited significantly higher phosphatase activity toward DiFMUP compared to the wild-type protein: the enzymatic turnover number (*k*_cat_)/half-maximum velocity (*K*_M_) value of the G95A mutant form was 9.3-fold higher than that of wild-type Tk-PTP at 20°C and 3.1-fold higher at 60°C (Yun et al., 2018). In contrast, although the P-loop (H95-C-V-G-G-I-G-R-T103) of SsoPTP contained the “GG” motif like Tk-PTP, it adopted a catalytically active conformation in all eight crystal structures, including the apo form (Fig. 3C, left top). The *k*_cat_ of SsoPTP, measured at pH 4.5 using *p*-NPP as a substrate, was 17-fold higher at 60°C (34.0 s^-1^) than at 25°C (2.0 s^-1^) (Pinkston et al., 2021). As these values are comparable to those of Tk-PTP (30.0 s^-1^ at 60°C and 4.74 s^-1^ at 20°C), determined at pH 4.5 using DiFMUP as a substrate (Yun et al., 2018), the enzymatic activity of SsoPTP is also considered to increase in a temperature-dependent manner. Therefore, a GG motif-associated conformational change in the P-loop may also occur in SsoPTP, which warrants further investigation.

Another distinguishing structural feature of archaeal DUSPs is the presence of dual general acid/base residues, similar to those observed in DUSP23a (Kuznetsov and Hengge, 2013). Structural and biochemical analyses revealed that, in addition to the highly conserved aspartate residue (Asp69 in SsoPTP and Asp63 in Tk-PTP), both archaeal DUSPs possess an alternative general acid/base residue (Pinkston et al., 2021; Yun et al., 2018): Glu40 in SsoPTP and Glu132 in Tk-PTP (Fig. 2B, right). In Tk-PTP, the *k*_cat_/*K*_M_ value of the D63N∙E132Q mutant protein (0.140 s^-1^ mM^-1^ at 20°C and 3.09 s^-1^ mM^-1^ at 60°C) was 47- and 35-fold lower than that of the wild-type protein (6.59 s^-1^ mM^-1^ at 20°C and 109 s^-1^ mM^-1^ at 60°C), and 2- and 4-fold lower than that of the D63N single mutant (0.255 s^-1^ mM^-1^ at 20°C and 11.6 s^-1^ mM^-1^ at 60°C) when tested against the DiFMUP substrate (Yun et al., 2018). These results suggest that Asp63 and Glu132 function as primary and supportive general acid/base residues, respectively, in Tk-PTP. In contrast, biochemical analyses using the *p*-NPP substrate showed that D69N (0.07 s^-1^ at 20°C and 0.24 s^-1^ at 60°C) and E40Q (0.05 s^-1^ at 20°C and 0.53 s^-1^ at 60°C) mutations each led to a substantial reduction in the *k*_cat_ value compared to that of the wild-type SsoPTP (2.0 s^-1^ at 20°C and 34.0 s^-1^ at 60°C), suggesting that Asp69 and Glu40 play critical roles in the dephosphorylating activity of SsoPTP (Pinkston et al., 2021).

Next, BLAST-based homology searches were performed to identify additional putative archaeal DUSP proteins. Tk-PTP homologues with higher sequence identity (59.0–84.4%) were identified in the genomes of 33 *Thermococcus*, two *Palaeococcus*, and three *Pyrococcus* species, belonging to the family *Thermococcaceae*, order *Thermococcales*, class *Thermococci*, and phylum *Methanobacteriota* (Table S2). Interestingly, 15 homologues of Tk-PTP with lower sequence identity (35.3–45.5%) were also found in the genomes of archaea from two additional phyla: 14 from the phylum *Thermoproteota* and one from the phylum *Nitrososphaerota* (Table S2), indicating that DUSPs are present in archaeal species from at least three different phyla. Notably, all of these proteins retained at least seven of the nine amino acids in the P-loop region (H-C-x-G-G-x-G-R-S) including the GG motif (Table S2), suggesting that the GG motif is a common feature of archaeal DUSPs.

Collectively, structural analyses revealed that archaeal DUSPs are characterized by hyperthermostability attributed to their dense interior protein packing, a GG motif thought to induce conformational changes in the catalytic P-loop region, and dual general acid/base residues. However, several questions remain unanswered, including their physiological substrates, the precise biological functions of archaeal DUSPs, and whether DUSPs are present in other archaeal lineages. Further studies are needed to resolve these questions.

### Structural analysis of viral DUSPs

PTPs are also present in various viruses (Guan and Dixon, 1993). To date, the only group of viral PTPs that has been extensively studied at the structural and biochemical levels is poxviral DUSPs, including *variola* virus H1 (PDB code: 2P4D), *vaccinia* virus VH1 (PDB codes: 2Q05, 2RF6, and 3CM3), *monkeypox* virus H1 (PDB code: 8GZ4), and *orf* virus OH1 (PDB code: 5NCR) (Cui et al., 2023; Koksal and Cingolani, 2011; Koksal et al., 2009; Mann et al., 2008; Najarro et al., 2001; Phan et al., 2007; Segovia et al., 2017) (Table 3). Poxviruses are large, enveloped, double-stranded DNA viruses that infect various vertebrate species, including cows, monkeys, and humans (Sprygin et al., 2022). They have been under intensive investigation because of their ability to cause several fatal diseases, such as smallpox and *monkeypox*, which are caused by *variola* and *monkeypox* viruses, respectively (Sossai et al., 2023). The poxvirus genome encodes approximately 200 proteins, about half of which are immunomodulatory proteins that interfere with host immune processes upon viral infection (Yu et al., 2021). Among them, DUSP proteins of poxviruses, including *vaccinia* virus (Koksal and Cingolani, 2011; Koksal et al., 2009; Najarro et al., 2001) and *variola* virus (Mann et al., 2008), dephosphorylate and impair nuclear import of STAT1, a key host immune regulator. This, in turn, downregulates interferon-γ signaling and the host antiviral response (Koksal et al., 2009; Mann et al., 2008; Najarro et al., 2001). Four crystal structures of poxviral DUSPs have been determined, all of which exhibit high structural similarity upon superimposition: *vaccinia* virus VH1 aligns closely with *variola* virus H1 (0.51 Å over 163 aligned residues), *monkeypox* virus H1 (0.58 Å over 164 aligned residues), and *orf* virus OH1 (1.07 Å over 144 aligned residues) (Fig. 4A, right). For this review, the crystal structure of VH1 from *vaccinia* virus (PDB code: 3CM3), a member of the *Orthopoxvirus* genus of the *Poxviridae* family, was selected as a representative model for poxviral DUSPs.

VH1 adopts a typical DUSP fold comprising a core region (α2–α5 and β1–β5) and mid-domain variation components α0 and α1' (Fig. 1C). A prominent structural feature of VH1 (and three other poxviral DUSPs) is that it forms a unique and stable domain-swapped dimer, in which the N-terminal α0 helix from one protomer interacts with the α4 and α5 helices of the opposing molecule (Fig. 4B, left). In-depth structural analysis revealed that this homodimer is stabilized by multiple hydrophobic interactions, involving Tyr7, Lys8, Leu11, Leu12, and Thr15 in α0 of one protomer, and Met135, Phe138, Leu139, Tyr142, Lys159, Arg160, Ile163, Val167, and Ile168 in α4 and α5 of the other protomer (Fig. 4B, right). This dimerization is further supported by an electrostatic interaction between Lys8 of one protomer and Glu164 of the other (Fig. 4B, right). Given that STAT1 transitions from an antiparallel to a parallel dimer upon phosphorylation (Wenta et al., 2008), domain-swapped dimerization of VH1 may facilitate recognition of the phosphorylated STAT1 homodimer as a substrate, thereby enabling interference with the host antiviral response. Indeed, biochemical analyses using the 3-O-methylfluorescein phosphate substrate showed that the catalytic activity of VH1 (*k*_cat_/*K*_M_ of 2.05 s^-1^ mM^-1^) was substantially reduced by dimerization-disrupting mutations at the N-terminal α0 helix (*k*_cat_/*K*_M_ of 0.80 s^-1^ mM^-1^) and was nearly abolished upon removal of this region (*k*_cat_/*K*_M_ of 0.017 s^-1^ mM^-1^), highlighting the critical role of VH1 dimerization in its catalytic function (Koksal and Cingolani, 2011). Structural analysis of VH1 revealed that it has canonical features of a DUSP protein. It contains a shallow active site pocket (Fig. 2A, panel VI) along with a catalytically active P-loop (H^109^-C-A-A-G-V-N-R-S^117^; Fig. 3D) and adopts a closed conformation (ASC1), in which the highly conserved Asp79 residue serves as a general acid/base (Fig. 2B, right). Further structural and biochemical studies are needed to identify and characterize other viral DUSPs.  

## Conclusion

This review presents a comprehensive structural analysis of human and various microbial DUSPs, derived from bacteria (*P. aeruginosa* TpbA), archaea (*T. kodakarensis* Tk-PTP and *S. solfataricus* SsoPTP), and viruses (poxviral DUSPs such as *vaccinia* virus VH1), based on their three-dimensional structures. These enzymes share several structural features, including a conserved DUSP fold (Fig. 1), shallow active site pocket (Fig. 2A), conserved aspartate residue that functions as a general acid/base (Fig. 2B), and signature active site motif known as the P-loop (H-C-x-x-G-x-x-R-S/T; Fig. 3). However, distinct structural and functional differences were also observed. TpbA, a key regulatory factor in *P. aeruginosa* biofilm formation, remains the only DUSP protein identified and structurally characterized in bacteria. Nevertheless, a second candidate is proposed in this review: a putative DUSP protein from *Ca. Chlorohelix. allophototropha* (RefSeq: WP_341469495.1). Tk-PTP and SsoPTP, two archaeal DUSP proteins whose structures have been determined, are characterized by their hyperthermostability (experimentally verified for Tk-PTP and strongly inferred for SsoPTP), the presence of a “GG” motif in the P-loop region, and the use of dual general acid/base residues in catalysis. Notably, the substrates and biological functions of these archaeal DUSPs have not been identified. Poxviral DUSPs, which dephosphorylate and inactivate STAT1, form a stable domain-swapped homodimer that facilitates recognition of the phosphorylated STAT1 homodimer. Given that DUSP proteins share a structural backbone with classical PTPs, exhibit low specificity among phosphorylated serine, threonine, and tyrosine residues, and are the only type of PTP found across all three domains of life (bacteria, archaea, and eukarya) as well as in viruses, they may represent the ancestral form of PTPs. Identification and structural characterization of additional DUSP proteins from bacteria, archaea, and viruses would help resolve this question. Further analyses would also broaden the understanding of dephosphorylation, which plays a critical role in diverse biological processes.

## Figures and Tables

**Table 1.** Catalytic domain structures of human MKP-type DUSP proteins

Protein	Alias	PDB code	References
DUSP1	MKP-1, VH1	6APX, 6D65, 6D66, 6D67	Gumpena et al. (2018a, 2018b)
DUSP2	PAC-1	1M3G^*^	Farooq et al. (2003)
DUSP4	MKP-2, VH2	3EZZ	Jeong et al. (2009)
DUSP5	VH3	2G6Z	Jeong et al. (2007)
DUSP6	MKP-3, Pyst1	1MKP	Farooq et al. (2001); Stewart et al. (1999)
DUSP7	MKP-X, Pyst2	4Y2E	Lountos et al. (2015a)
DUSP8	VH5	4JMK	Jeong et al. (2014)
DUSP9	MKP-4, Pyst3	2HXP, 3LJ8	Almo et al. (2007); Jeong et al. (2011)
DUSP10	MKP-5	1ZZW, 2OUD, 6MC1, 7U4O, 7U4R, 7UMU, 7UMV, 7UN0, 7UN4, 7Y4B, 7Y4C, 7Y4D, 7Y4E	Gannam et al. (2020, 2022); Jeong et al. (2006b); Tao and Tong (2007); Zhang et al. (2011)
DUSP14	MKP-6	2WGP	Lountos et al. (2009)
DUSP16	MKP-7	4YR8	Liu et al. (2016); Zhang et al. (2011)

* Structure determined by nuclear magnetic resonance; all other structures were determined by X-ray crystallography.

**Table 2.** Catalytic domain structures of human atypical DUSP proteins

Protein	Alias	PDB code	References
DUSP3	VHR	1J4X, 1VHR, 3F81, 8TK2, 8TK3, 8TK4, 8TK5, 8TK6, 9DJ9	Schumacher et al. (2002); Wu et al. (2009, 2025); Yuvaniyama et al. (1996)
DUSP11	PIR1	4JMJ, 4MBB, 4NYH	Jeong et al. (2014); Sankhala et al. (2014)
DUSP12	YVH1	4JNB, 4KI9	Jeong et al. (2014)
DUSP13a	BEDP	5XJV	Wei et al. (2018)
DUSP13b	SKRP4, TMDP	2GWO, 2PQ5	Kim et al. (2007)
DUSP15	VHY	1YZ4	Yoon et al. (2005)
DUSP18	DUSP20	2ESB	Jeong et al. (2006a)
DUSP19	DUSP17, SKRP1	3S4E, 4D3P, 4D3Q, 4D3R	Jeon et al. (2015); Wei et al. (2011)
DUSP21	-	(none)	(none)
DUSP22	JSP1, VHX	1WRM, 4WOH, 6L1S, 6LMY, 6LOT, 6LOU, 6LVQ, 7C8S	Lai et al. (2020); Lountos et al. (2015b); Yokota et al. (2007)
DUSP23a	VHZ, DUSP25	2IMG, 4ERC	Agarwal et al. (2008); Kuznetsov et al. (2012)
DUSP23b	PTPMT1, MOSP	(none)	(none)
DUSP26	DUSP24, SKRP3	2E0T, 4B04, 4HRF, 5GTJ	Lokareddy et al. (2013); Won et al. (2013, 2016)
DUSP28	VHP	5Y15, 5Y16	Ku et al. (2017)
DUSP29	DUSP27. DUPD1	2Y96	Lountos et al. (2011)

All the structures were determined by X-ray crystallography. The word “none” indicates structures that have not yet been elucidated.

**Table 3.** Structures of microbial DUSP proteins

Classification	Species	Protein	PDB code	References
Bacteria	*P. aeruginosa*	TpbA	2M3V^*^, 4R0S, 4R0T	Koveal et al. (2013); Xu et al. (2015)
Archaea	*T. kodakarensis*	Tk-PTP	5Z59, 5Z5A, 5Z5B	Yun et al. (2018)
	*S. solfataricus*	SsoPTP	2DXP, 2I6I, 2I6J, 2I6M, 2I6O, 2I6P, 7MPC, PMPD,	Chu and Wang (2007); Pinkston et al. (2021)
Virus	Variola virus	H1	2P4D	Phan et al. (2007)
	Vaccinia virus	VH1	2Q05, 2RF6, 3CM3	Koksal et al. (2009)
	Monkeypox virus	H1	8GZ4	Cui et al. (2023)
	Orf virus	OH1	5NCR	Segovia et al. (2017)

* Structure determined by nuclear magnetic resonance; all other structures were determined by X-ray crystallography.

**Table 4.** Numbers of side chain-mediated intramolecular carbon−carbon contacts in human and archaeal DUSPs

Protein	PDB code	Residues	Intramolecular C−C contacts (< 4.5 Å)
DUSP3	1J4X	8–185	542 (3.04 / 1 residue)
DUSP28	5Y15	12–159	439 (2.97 / 1 residue)
Tk-PTP	5Z5A	1–147	632 (4.30 / 1 residue)
SsoPTP	2I6I	1–161	633 (3.93 / 1 residue)

## Supplementary Information

Table S1. List of TpbA homologues in bacteria

Table S2. List of Tk-PTP homologues in archaea
